# Identification of New Agonists and Antagonists of the Insect Odorant Receptor Co-Receptor Subunit

**DOI:** 10.1371/journal.pone.0036784

**Published:** 2012-05-08

**Authors:** Sisi Chen, Charles W. Luetje

**Affiliations:** Department of Molecular and Cellular Pharmacology, University of Miami Miller School of Medicine, Miami, Florida, United States of America; German Institute for Human Nutrition, Germany; Department of Molecular and Cellular Pharmacology, University of Miami Miller School of Medicine, Miami, Florida, United States of America

## Abstract

**Background:**

Insects detect attractive and aversive chemicals using several families of chemosensory receptors, including the OR family of olfactory receptors, making these receptors appealing targets for the control of insects. Insect ORs are odorant-gated ion channels, comprised of at least one common subunit (the odorant receptor co-receptor subunit, Orco) and at least one variable odorant specificity subunit. Each of the many ORs of an insect species is activated or inhibited by an unique set of odorants that interact with the variable odorant specificity subunits, making the development of OR directed insect control agents complex and laborious. However, several N-,2-substituted triazolothioacetamide compounds (VUAA1, VU0450667 and VU0183254) were recently shown to act directly on the highly conserved Orco subunit, suggesting that broadly active compounds can be developed. We have explored the chemical space around the VUAA1 structure in order to identify new Orco ligands.

**Principal Findings:**

We screened ORs from several insect species, using heterologous expression in *Xenopus* oocytes and an electrophysiological assay, with a panel of 22 compounds structurally related to VUAA1. By varying the nitrogen position in the pyridine ring and altering the moieties decorating the phenyl ring, we identified two new agonists and a series of competitive antagonists. Screening smaller compounds, similar to portions of the VUAA1 structure, also yielded competitive antagonists. Importantly, we show that Orco antagonists inhibit odorant activation of ORs from several insect species. Detailed examination of one antagonist demonstrated inhibition to be through a non-competitive mechanism.

**Conclusions:**

A similar pattern of agonist and antagonist sensitivity displayed by Orco subunits from different species suggests a highly conserved binding site structure. The susceptibility to inhibition of odorant activation by Orco antagonism is conserved across disparate insect species, suggesing the ligand binding site on Orco as a promising target for the development of novel, broadly active insect repellants.

## Introduction

Olfaction drives many insect behaviors, including those deleterious to human health. Insects detect attractive and aversive chemicals using several families of chemosensory receptors, including the OR family of insect olfactory receptors [1,2,3]. These receptors, located on the dendrites of olfactory sensory neurons (OSNs), are appealing targets for the control of insects involved in disease propagation and agricultural damage. In contrast to mammalian ORs, which are a large family of G-protein coupled receptors, the insect ORs are a novel class of ligand (odorant) gated, non-selective cation ion channels [4,5]. Insect ORs are multimeric complexes of unknown stoichiometry, formed by a common subunit (the odorant receptor co-receptor subunit known as Orco [6]) that is highly conserved across different species and a variable subunit that confers odorant specificity [3,7,8,9,10,11,12,13]. These receptors have generally been thought to function as obligate heteromultimers [3], with only a few reports of homomeric function [5,14,15]. Within an individual OR, both Orco and the specificity subunit may make contributions to the structure and properties of the ion pore [16,17,18]. The specificity subunits are thought to mediate odorant recognition, because changing this subunit can alter odorant preference [19,20,21,22] and mutations in a specificity subunit can alter odorant sensitivity [23,24]. Because Orco is common to every insect OR, the great diversity in odorant preference among the ORs of each insect species is generated by the specificity subunits [3].

The novel structure of insect ORs and lack of similar receptors in humans and other mammals [7] suggests that improved control of destructive insect species can be achieved through the development of new, OR directed compounds with higher selectivity and lower environmental toxicity than currently available insecticides and repellants. One approach to developing these compounds involves the identification of particular specificity subunits that mediate recognition of behaviorally specific odorants [19,22,25,26,27], followed by extensive ligand screening [28]. A drawback of this approach is that high diversity among the specificity subunit repertoires of different species and variation in which odorants and specificity subunits are key to species specific behaviors [29] means that receptor identification, extensive screening and ligand optimization would be required for each of the many potential target receptors. Compounds that are active at multiple ORs of many different species would be of much greater utility. The identification of VUAA1 [28] as a novel agonist and VU0183254 as a novel antagonist of insect ORs [30], each acting at a binding site on the Orco subunit from several insect species, suggests that such generally active compounds can be developed.

In this study, we expand the repertoire of Orco agonists and antagonists. We screen ORs from several insect species, using heterologous expression in *Xenopus* oocytes and electrophysiological recording, with a panel of compounds structurally related to VUAA1. We identify two new Orco agonists, as well as a series of competitive antagonists of the Orco subunit. A similar pattern of agonist and antagonist sensitivity displayed by Orco subunits from a variety of different species suggests that the binding site on Orco has a highly conserved structure. We also show that the Orco antagonists can inhibit odorant activation of insect ORs through a non-competitive mechanism. Susceptibility to inhibition of odorant activation through Orco antagonism is conserved across disparate insect species, suggesting Orco pharmacology as a promising area for the development of novel, broadly active insect repellants.

## Results

**Figure 1:**
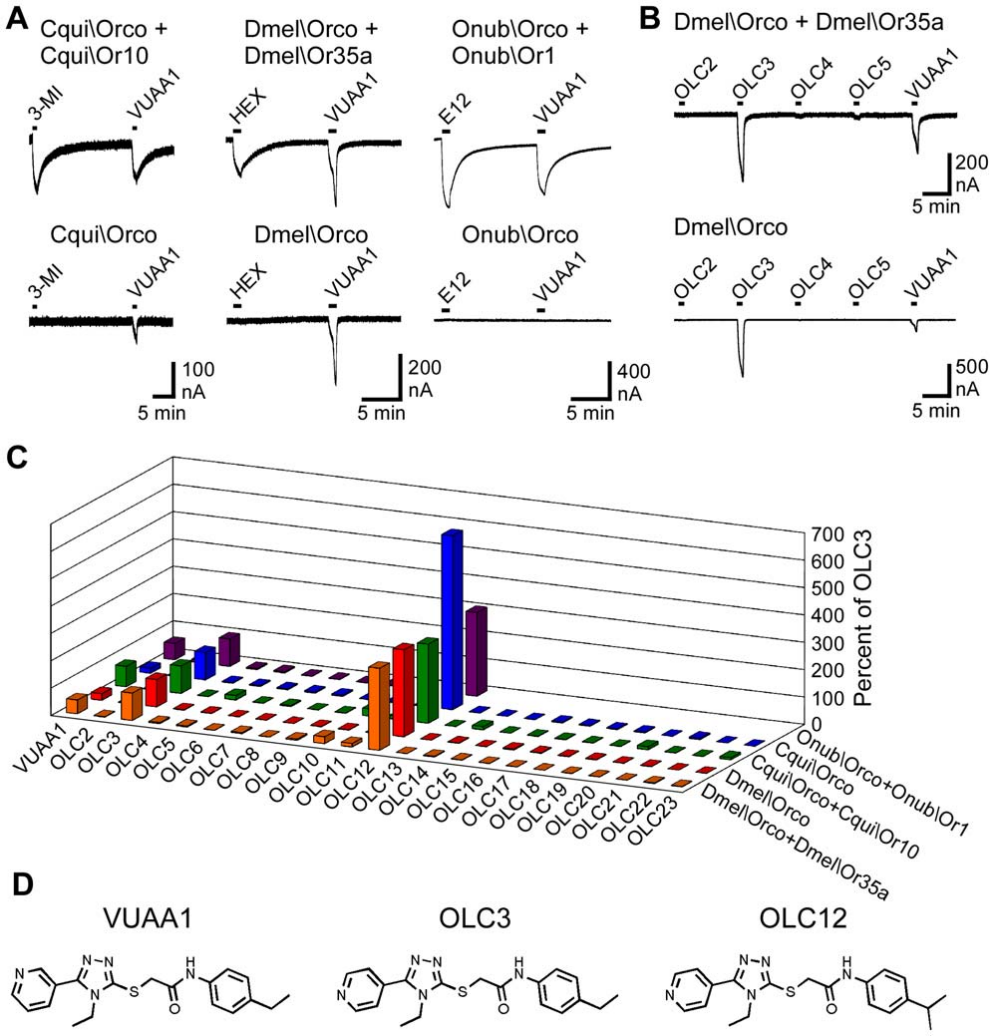
Identification of OLC3 and OLC12 as Orco agonists. **A**) VUAA1 activates both heteromeric (top traces) and homomeric (bottom traces) insect ORs expressed in *Xenopus* oocytes and assayed by two electrode voltage clamp electrophysiology. Compounds were applied for 60 sec with a 9 min wash between applications. VUAA1 was applied at 100 µM. Oocytes expressing Cqui\Orco + Cqui\Or10 or Cqui\Orco were also exposed to 30 nM 3-methylindole (3-MI). Oocytes expressing Dmel\Orco + Dmel\Or35a or Dmel\Orco were also exposed to 3 µM Hexanol (HEX). Oocytes expressing Onub\Orco + Onub\Or1 or Onub\Orco were also exposed to 1 µM E12–14:OAc (E12). **B)** OLC3 activates both heteromeric and homomeric insect ORs. Oocytes expressing Dmel\Orco + Dmel\Or35a (top trace) or Dmel\Orco (bottom trace) were challenged with 60 sec applications of 100 µM OLC2, OLC3, OLC4, OLC5 and VUAA1, with 9 min washes between applications. **C**) Results from a screen of 22 compounds (each applied at 100 µM) for Orco agonist activity. Responses are normalized to the response of the same oocyte to 100 µM OLC3 and presented as the mean of 3–8 oocytes (SEM values may be found in Table S1). **D**) Structures of VUAA1, OLC3 and OLC12.

In Figure 1A, we expressed ORs from several insect species in *Xenopus* oocytes and assayed receptor function by two-electrode voltage clamp electrophysiology: Cqui\Orco+Cqui\Or10, an OR from the Southern House Mosquito (*Culex quinquefasciatus*) that responds to oviposition attractants, such as 3-methylindole [25]; Dmel\Orco+Dmel\Or35a, an OR from *Drosophila melanogaster* that responds to a variety of aliphatic alcohols, aldehydes and esters [16,20]; Onub\Orco+Onub\Or1, an OR from the European Corn Borer (*Ostrinia nubilalis*) that responds to mating pheromones, such as E12–14:OAc [31]. Similar to the previous report [28], we found that VUAA1 (100 µM) could activate each of these ORs. Also as reported previously [28], we found that Orco could form a functional channel when expressed in the absence of specificity subunits. We observed current responses when VUAA1 was applied to oocytes expressing either Dmel\Orco or Cqui\Orco, but no specificity subunits (Fig. 1A). We did not observe functional expression of Onub\Orco channels, but whether this is due to a failure of expression or a failure of function is not known. No responses to VUAA1 were observed with sham (water) injected oocytes (Fig. S1).

**Figure 2:**
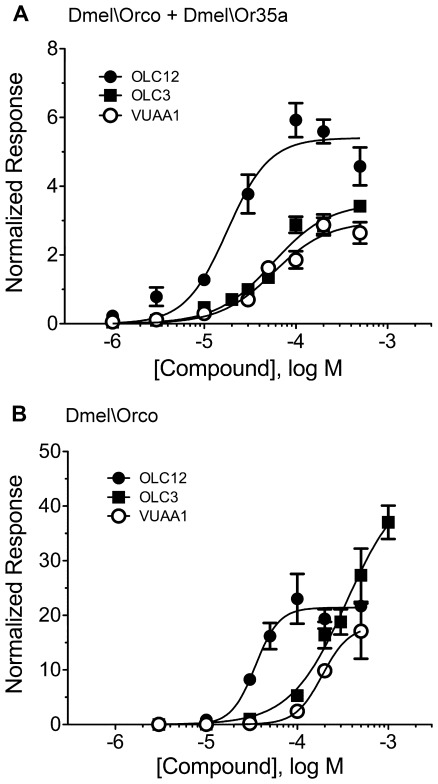
OLC12 is a more potent Orco agonist than VUAA1 or OLC3. Concentration-response analysis for VUAA1, OLC3 and OLC12 activation of Dmel\Orco + Dmel\Or35a (**A**) and Dmel\Orco (**B**). Each response was normalized to the response of the same oocyte to 30 µM OLC3. EC_50_ values can be found in Supplementary Table 2.

We sought to identify additional agonists of the Orco subunit by screening a panel of 22 compounds (termed Orco Ligand Candidates, OLCs). Fourteen compounds (OLC2–OLC15) were structurally related to the full VUAA1 structure (Fig. S2), with alterations to the pyridine ring (OLC2–OLC4) and a variety of substitutions on the phenyl ring (OLC5 -OLC15). Eight compounds (OLC16–OLC22) were similar to portions of the VUAA1 structure (Fig. S3). All 22 OLCs were screened at a concentration of 100 µM against oocytes expressing Dmel\Orco+Dmel\Or35a, Dmel\Orco alone, Cqui\Orco+Cqui\Or10 or Cqui\Orco alone, and a subset (OLC2–9, OLC12) was screened against oocytes expressing Onub\Orco+Onub\Or1 (Fig. 1B,C, Table S1). While most of these compounds displayed little or no agonist activity, the screen identified two new agonists of the Orco subunit (Fig. 1D). OLC3, with the pyridine nitrogen moved from the 3 position (as in VUAA1) to the 4 position, was effective in activating each of the 3 receptor complexes, as well as the homomeric channels formed by Dmel\Orco and Cqui\Orco. OLC12, with the nitrogen in the 4 position of the pyridine ring and the 4-ethyl moiety on the phenyl ring (as in VUAA1) changed to a 4-isopropyl moiety, was more effective than either VUAA1 or OLC3 in activating the 3 receptor complexes and the homomeric channels formed by Dmel\Orco and Cqui\Orco. No responses to OLC3 or OLC12 were observed with sham (water) injected oocytes (Fig. S1). We explored the activity of these agonists in more detail by constructing concentration-response curves for activation of Dmel\Orco+Dmel\Or35a (Fig. 2A) and Dmel\Orco alone (Fig. 2B). OLC12 was significantly more potent than VUAA1 and OLC3 in both the heteromeric and homomeric contexts (Table 1). OLC 12 also displayed a significantly greater maximal response in the Dmel\Orco+Dmel\Or35a context (Fig. 2A), while OLC3 appeared to yield a greater maximal response in the Dmel\Orco context (Fig. 2B). However, we were unable to obtain saturation for OLC3 (or VUAA1) activation of Dmel\Orco alone, because these compounds were not fully soluble at higher concentrations.

**Table 1:** EC_50_ and IC_50_ values for agonists and antagonists of Dmel\Orco.

Compound	EC_50_ (µM)		IC_50_ (µM)	
	Dmel\Orco + Dmel\Or35a	Dmel\Orco	Dmel\Orco + Dmel\Or35a (activated by 10 µM OLC12)	Dmel\Orco (activated by 30 µM OLC12)
VUAA1	57**±**13	190±30		
OLC3	56±8	346±126		
OLC12	18±3*** †	35±3*** †††		
OLC2			24±5	23±3
OLC9			40±9	69±7
OLC14			81±23	25±4
OLC15			15±1	15±2
OLC20			55±15	57±19
OLC22			26±4	52±18

Statistical differences from OLC3:

*** p<0.001. Statistical differences from VUAA1:

† p<0.05;

††† p<0.001.

**Figure 3:**
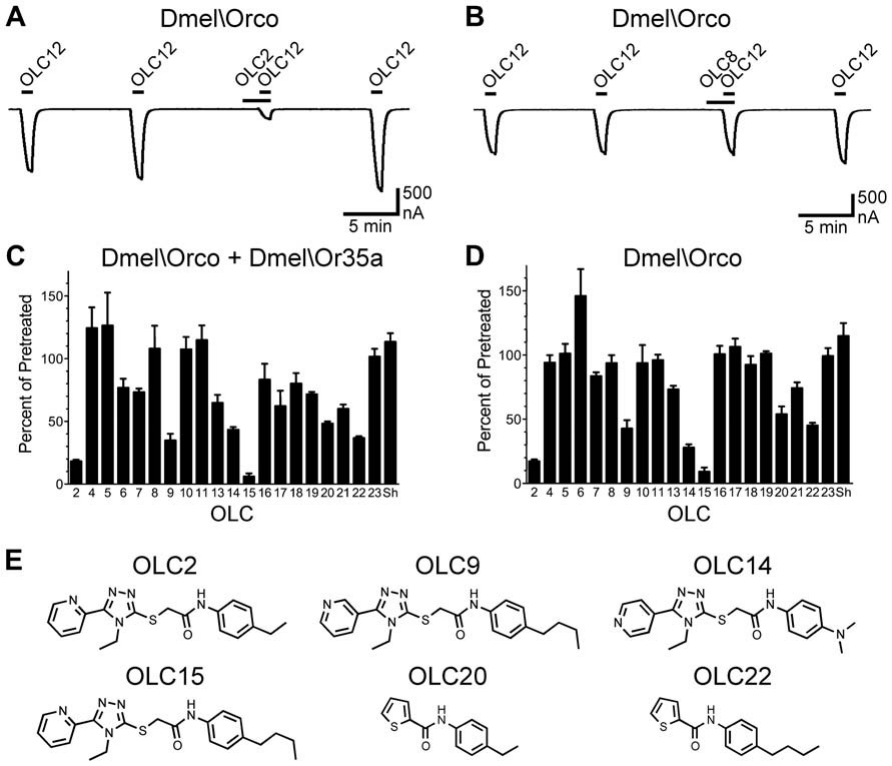
Identification of new Orco antagonists. **A**) Co-application of 100 µM OLC2 inhibits activation of Dmel\Orco by 30 µM OLC12. **B**) Co-application of 100 µM OLC8 fails to block activation of Dmel\Orco by 30 µM OLC12. **C–D**) Results of a screen of 20 compounds for Orco antagonism. Responses of Dmel\Orco + Dmel\Or35a to 10 µM OLC12 (EC_25_) (**C**) or of Dmel\Orco to 30 µM OLC12 (EC_39_) (**D**) in the presence of 100 µM of each candidate antagonist are presented as a percentage of the average of the two preceding responses to OLC12 alone (mean±SEM, n = 3−6). Sh, sham. **E**) Structures of Orco antagonists.

**Figure 4:**
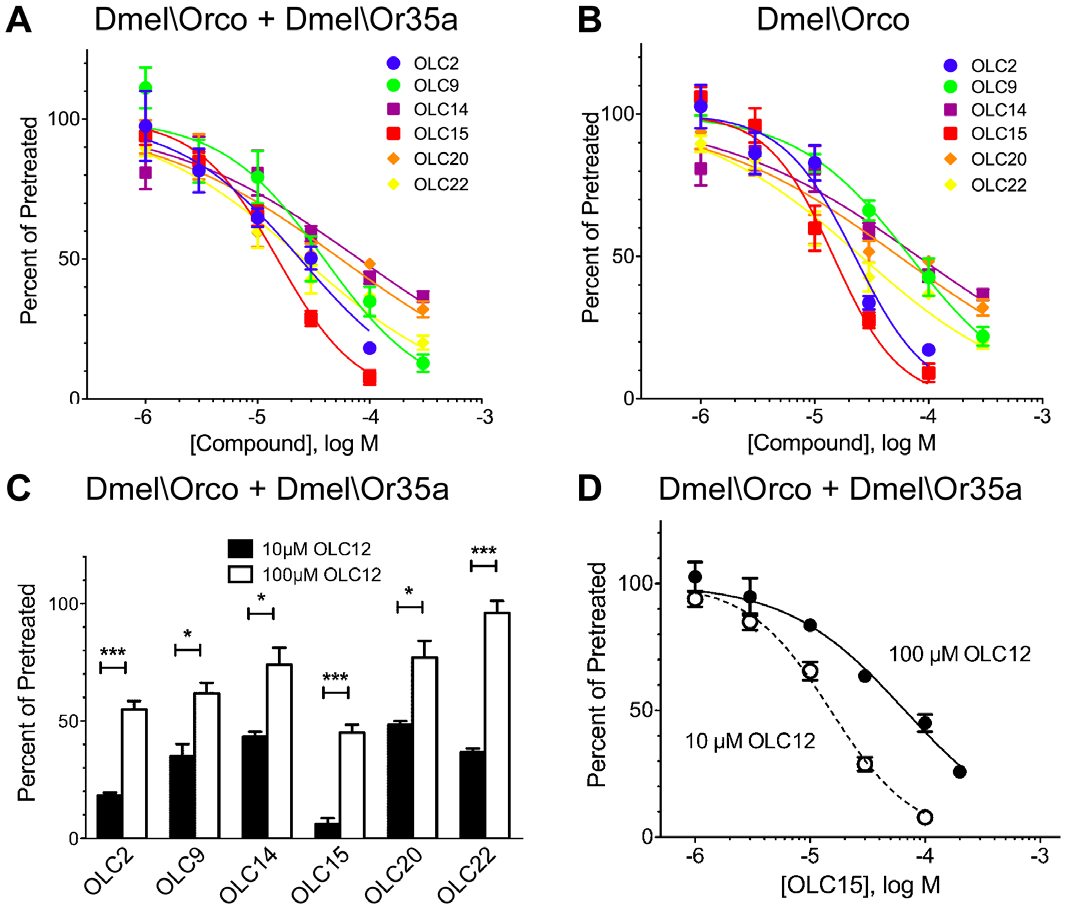
Competitive antagonism of Orco activation. **A-B**) Concentration-inhibition analysis for OLC2, OLC9, OLC14, OLC15, OLC20 and OLC22 inhibition of Dmel\Orco + Dmel\Or35a activated by 10 µM OLC12 (EC_25_) (**A**) and Dmel\Orco activated by 30 µM OLC12 (EC_39_) (**B**). IC_50_ values may be found in Table 1. **C**) Increasing the concentration of agonist (OLC12) decreases the effectiveness of Orco antagonists. 100 µM OLC2, OLC9, OLC14, OLC15, OLC20, or OLC22 was co-applied with 10 µM OLC12 or 100 µM OLC12 to oocytes expressing Dmel\Orco + Dmel\Or35a. Responses in the presence antagonist are presented as a percentage of the average of the two preceding responses to OLC12 alone (mean±SEM, n = 3−5). Statistical significance (t-test): *p<0.05; **p<0.01; ***p<0.001. **D**, Altering agonist (OLC12) concentration, shifts the OLC15 inhibition curve. The IC_50_ values for OLC15 inhibition of 10 µM OLC12 (15±1 µM) and 100 µM OLC12 (67±7 µM) are significantly different (p<0.0001, F test).

We next screened the panel for antagonist activity. OLC12 was used to activate Dmel\Orco+Dmel\Or35a (10 µM OLC12, the EC_25_) or Dmel\Orco (30 µM OLC12, the EC_39_) and 100 µM of each compound (the 20 remaining compounds after OLC3 and OLC12 were excluded) was tested for the ability to inhibit the OLC12 response (Fig. 3). While many of these compounds showed some antagonist activity, we chose to concentrate on six compounds that displayed substantial inhibition of agonist activation of both Dmel\Orco+Dmel\Or35a and Dmel\Orco alone (Fig. 3E): OLC2, with the pyridine nitrogen shifted to the 2 position; OLC9, with a 4-butyl moiety on the phenyl ring; OLC15, with both the pyridine nitrogen in the 2 position and a 4-butyl moiety on the phenyl ring; OLC14, with the pyridine nitrogen at the 4 position and a dimethylamino moiety on the phenyl ring; OLC20, with a structure similar to a portion of the OLC2; OLC22, similar to a portion of the OLC9 structure. We examined the activity of these compounds in more detail by constructing concentration-inhibition curves for Dmel\Orco+Dmel\Or35a and Dmel\Orco alone (Fig. 4A–B, Table 1). These compounds displayed similar potencies for inhibition of OLC12 activation, with IC_50_’s ranging from 15 µM to 81 µM. It is important to note that while the IC_50_ values for antagonism by these compounds may be directly compared within one receptor context (Dmel\Orco+Dmel\Or35a or Dmel\Orco alone), caution should be exercised when comparing values for Dmel\Orco+Dmel\Or35a with those for Dmel\Orco alone. The agonist (OLC12) concentrations used in each case were of similar potency (EC_25_ and EC_39_, respectively), but were not precisely equipotent. When these six compounds, as well as OLC8 and OLC16, were tested against Cqui\Orco+Cqui\Or10 and Cqui\Orco (Fig. S4), the observed pattern of antagonist activity was similar to what we observed for Dmel\Orco+Dmel\Or35a and Dmel\Orco.

The antagonists that we have identified have structural similarity to VUAA1, OLC3 and OLC12, suggesting that they may compete for occupation of the same site on the Orco subunit. To determine whether this is the case, we compared the extent of blockade of Dmel\Orco+Dmel\Or35a that could be achieved by 100 µM of each of six antagonists (OLC2, OLC9, OLC14, OLC15, OLC20 and OLC22) when the agonist (OLC12) concentration was increased from 10 µM (as in Fig. 3C) to 100µM (Fig. 4C). In each case, the antagonist was significantly less effective at inhibiting the response to the higher concentration of agonist, indicating a competitive interaction. We examined this issue in more detail for OLC15 antagonism of OLC12 activation of Dmel\Orco+Dmel\Or35a (Fig. 4D). Concentration-inhibition analysis of OLC15 inhibition of activation by 10 µM OLC12 yielded an IC_50_ of 15±1 µM. When the concentration of OLC12 was increased to 100 µM, the concentration-inhibition curve was significantly shifted to the right (IC_50_ = 67±7 µM, p<0.0001, F test), again indicating a competitive interaction.

**Figure 5:**
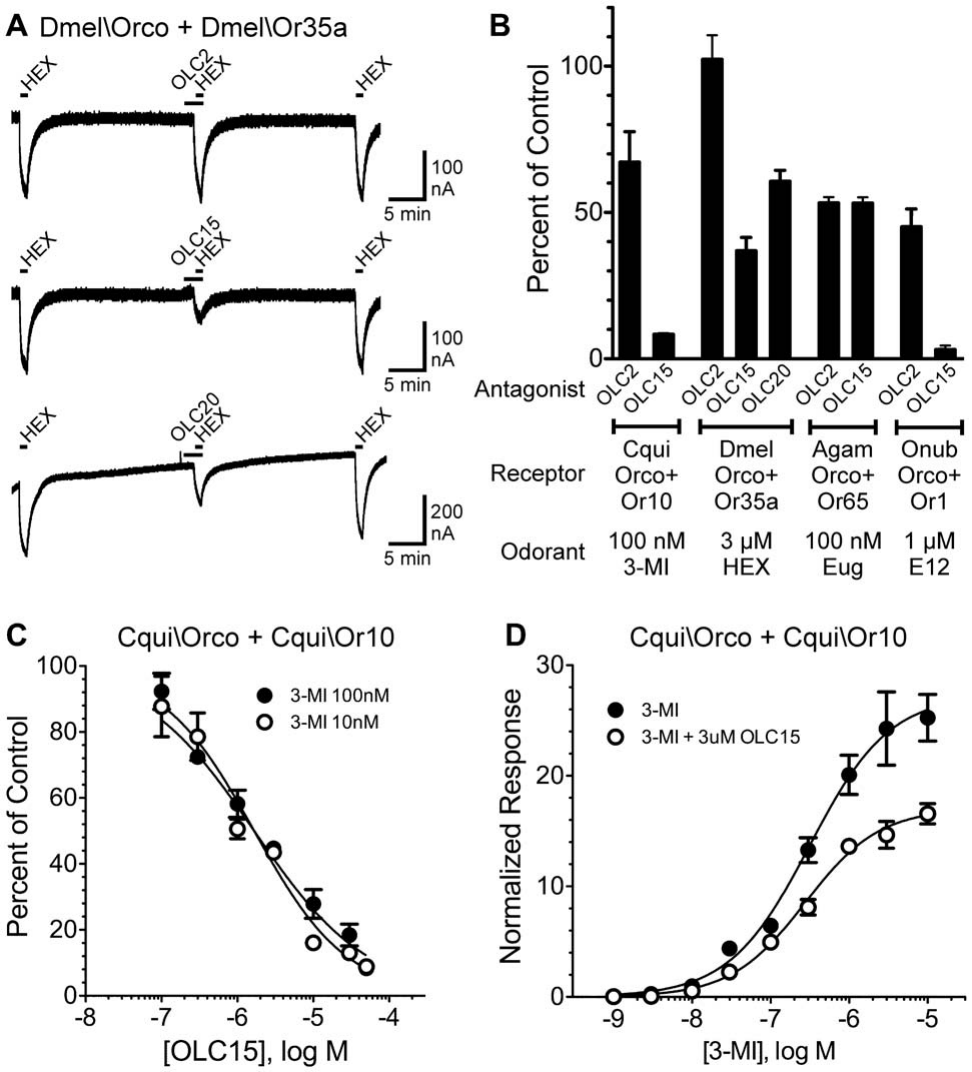
Non-competitive inhibition of odorant activation of insect ORs by an Orco antagonist. **A**) Oocytes expressing Dmel\Orco + Dmel\Or35a were exposed to 60 sec applications of 3 µM hexanol (HEX), with 20 min washes between applications. OLC2, OLC15 or OLC20 (100 µM) were applied for 90 sec preceding the second application of HEX and then co-applied during the HEX application. *Top trace*, OLC2 fails to inhibit HEX activation of Dmel\Orco + Dmel\Or35a. *Middle trace*, OLC15 inhibits HEX activation of Dmel\Orco + Dmel\Or35a. *Bottom trace*, OLC20 inhibits HEX activation of Dmel\Orco + Dmel\Or35a. **B**) OLC compounds inhibit odorant activation of ORs from a variety of insect species. Oocytes expressing Cqui\Orco+Cqui\Or10 were activated by 100 nM 3-methylindole (3-MI), oocytes expressing Dmel\Orco + Dmel\Or35a were activated by 3 µM HEX, oocytes expressing Agam\Orco + Agam\Or65 were activated by 100 nM eugenol (Eug) and oocytes expressing Onub\Orco + Onub\Or1 were activated E12–14:OAc (E12). Current responses in the presence of OLC compounds (100 µM) were compared to the preceding response to odorant alone and are presented as mean±SEM (n = 3−14). **C**) Altering odorant concentration fails to alter the inhibition curve for OLC15 antagonism of Cqui/Orco + Cqui\Or10 activation by 3-MI. The IC_50_ values for OLC15 inhibition of responses to 10 nM 3-MI (1.5±0.2 µM) and 100 nM 3-MI (2.0±0.3 µM) do not differ (p = 0.13, F test). **D**) Co-application of 3 µM OLC15 significantly reduces the maximal response to 3-MI, but does not alter the EC_50_ for 3-MI activation. The maximal response to 3-MI in the presence of 3 µM OLC15 was significantly lower (62±2%) than the maximal response in the absence of OLC15 (p<0.05, F test). The EC_50_ for 3-MI activation of Cqui\Orco + Cqui\Or10 was 313±103 nM, while the EC_50_ in presence of 3 µM OLC15 was 275±41 nM (p = 0.81, F test).

The identification of compounds, such as OLC15, that competitively antagonize the activation of Orco by agonists such as OLC3 and OLC12 is interesting. However, our current lack of knowledge about a physiological role for Orco agonism makes the utility of antagonizing of this process questionable. Thus, we wondered whether these Orco antagonists could also interfere with odorant activation. A recent publication [30] identified VU0183254 as an Orco antagonist capable of allosterically inhibiting odorant activation of ORs from *A. gambiae*. We tested whether a similar effect could be exerted by OLC2, OLC15 and OLC20 on the response of Dmel\Orco+Dmel\Or35a to hexanol. While OLC2 (100 µM) did not inhibit the hexanol response, co-application of OLC15 (50 µM) or OLC20 (200 µM) resulted in substantial inhibition (Fig. 5A,B). We then tested OLC2 and OLC15 against ORs from several additional species (Fig. 5B). Both compounds inhibited activation of Cqui\Orco+Cqui\Or10 by 3-methylindole, activation of Agam\Orco+Agam\Or65 by eugenol and activation of Onub\Orco+Onub\Or1 by E12–14:OAc. The *C. quinquefasciatus* and *O. nubilalis* ORs were significantly more sensitive to inhibition by OLC15 than OLC2 (p<0.01, t-test), while the *A. gambiae* OR was equally sensitive to both compounds. Thus, while there was variation in sensitivity, odorant activation of each OR could be inhibited through Orco antagonism.

To further understand the mechanism of inhibition of odorant activation by Orco antagonism, we examined the effect of OLC15 on Cqui\Orco+Cqui\Or10 in more detail. Concentration-inhibition curves for OLC15 were generated at two different concentrations of 3-methylindole. The EC_50_ for activation of Cqui\Orco+Cqui\Or10 by 3-methylindole is 90±17 nM [25]. The IC_50_ values for OLC15 inhibition of receptor activation by 10 nM 3-methylindole (EC_10_) and 100 nM 3-methylindole (EC_53_) were not significantly different (Fig. 5C), suggesting a non-competitive mechanism for OLC15 inhibition of odorant activation. This contrasts with the competitive mechanism for OLC15 inhibition of direct Orco activation by OLC12 that we demonstrated with a *Drosophila* receptor (Fig. 4C,D). Comparison of the concentration-response relationship for 3-methylindole activation of Cqui\Orco+Cqui\Or10 in the absence and presence of OLC15 showed that while the maximal response was significantly reduced by the presence of OLC15, the EC_50_ for activation was unchanged (Fig. 5D). This result provides further support for a non-competitive mechanism for inhibition of odorant activation by Orco antagonists.

## Discussion

The recent identification of VUAA1 as an activator of insect ORs through direct Orco agonism [28] and a subsequent report identifying VU0183254 as an Orco antagonist [30], have revealed the existence of a ligand-binding site on the Orco subunit. We have expanded on these findings by identifying two additional Orco agonists, as well as a larger series of Orco antagonists. With this expanded panel of agonists and antagonists, we compared the pharmacological properties of Orco subunits from several different species. We found a similar pattern of activation and inhibition by these compounds among Orco subunits of disparate insect species. This result suggests that the ligand-binding site on Orco is structurally conserved, consistent with the high level of protein sequence identity among Orco subunits of different species [8,9]. The *in vivo* function of this site, in any, is currently unknown.

While much additional work will be required, our results, together with the recent reports from Zwiebel and colleagues [28,30], allow an initial discussion of the structural requirements for agonism and antagonism of the Orco subunit of insect ORs. The requirements for agonist activity appear to be quite strict, with only minor changes being tolerated. Movement of the pyridine nitrogen from the 3 position to the 4 position and addition of a methyl to create an isopropyl group on the phenyl ring both yield compounds with agonist activity (OLC3 and OLC12). The three compounds with substantial agonist activity (VUAA1, OLC3 and OLC12) vary in both potency (EC_50_) and efficacy (maximal response). In a whole receptor context (Dmel\Orco+Dmel\Or35a), OLC12 displays greater efficacy than OLC3 and VUAA1, defining the latter two compounds as partial agonists. However, in an Orco alone context (Dmel\Orco), OLC3 appears to have the greater efficacy (although solubility issues prevent us from achieving saturation), with OLC12 and VUAA1 being partial agonists.

All other modifications to the VUAA1 lead structure yielded compounds that were, with a few exceptions (OLC10 and OLC11), devoid of agonist activity. The very low level of agonist activity observed with OLC10, with the ethyl group on the phenyl ring changed to a bromo group, is converted to competitive antagonism with the addition of a methyl group at the 2 position of the phenyl ring to yield VU0450667 [30]. While most of the compounds tested lack agonist activity, many displayed antagonist activity. Minor modifications, such as the shift of the pyridine nitrogen to the 2 position (OLC2) or conversion of the 4-ethylphenyl group to a 3,4-dimethylphenyl group (OLC13), eliminated agonist activity and generated antagonists. Larger modifications to the phenyl group, such as conversion to a 4-butylphenyl group (OLC15) or a 4-dimethylaminophenyl group (OLC14), also produced antagonists. VU0183254, a related structure with a furanotriazole core and a phenothiazine substituting for the 4-ethylphenyl group, also functions as an antagonist [30]. The antagonist activity of VU0183254 [30], as well as the smaller structures we have identified (OLC20 and OLC22), suggests that the chemical space containing viable Orco antagonists may be relatively broad and that further exploration is merited.

The observation that Orco subunits can form functional channels in the absence of specificity subunits ([5,28,30] and this study) suggests that both heteromeric and homomeric channel complexes may exist in oocytes when we express a specificity subunit and an Orco subunit. This may raise concerns about whether Orco ligands can act on heteromeric complexes. The three agonists (VUAA1, OLC3 and OLC12) and six antagonists (OLC2, OLC9, OLC14, OLC15, OLC20 and OLC22) we examined in detail were not significantly more potent when applied to oocytes expressing both Dmel\Or35a and Dmel\Orco than when applied to oocytes expressing Dmel\Orco alone (Table 1). However, Orco agonists (VUAA1, OLC3 and OLC12) and antagonists (OLC2 and OLC15) clearly interact with a heteromeric Onub\Orco+Onub\Or1 (Figs. 1 and 5), because homomeric Onub\Orco is non-functional (Fig. 1). Further, the Orco antagonists we have identified were capable of inhibiting odorant activation of heteromeric ORs (Fig. 5). Similar results were reported for the antagonist VU0183254 [30]. These findings indicate that the binding site of the Orco subunit is functional when Orco is part of a heteromeric OR complex.

Interestingly, Orco antagonists vary in their ability to allosterically inhibit activation of ORs by odorant. OLC2 and OLC15 display similar potencies for inhibition of direct Orco activation by an Orco agonist such as OLC12 (Fig. 4A,B). However, while OLC15 can also inhibit odorant activation of the *Drosophila* receptor, OLC2 could not (Fig. 5B). It is possible that OLC2 inhibition of odorant activation of this receptor may become apparent at higher concentrations, but the poor solubility of OLC2 at concentrations above 100 µM renders this problematic. A differential ability of Orco antagonists to inhibit odorant activation was also observed with ORs from *A. gambiae* [30]. Thus, relative potencies for inhibition of direct Orco activation (by an Orco agonist) may not be good predictors of the ability of a compound to allosterically inhibit activation of the receptor by odorant.

The presence of a conserved binding site through which ORs from many different insect species may be activated or inhibited obviates the need for the laborious development of compounds directed toward particular ORs of individual species. By targeting Orco, the development of compounds with simultaneous utility against many insect species may be possible. Indeed, in our study and the previous reports [28,30], Orco agonism and antagonism was observed with ORs of insect species from several different orders, including Diptera (*D. melanogaster*, *A. gambiae*, *C. quinquefasciatus*), Lepidoptera (*O. nubilalis*, *H. virescens*) and Hymenoptera (*H. saltator*). An OR from *A. aegypti* has also been shown to respond to VUAA1 and OLC3 [32]. This conservation of susceptibility to Orco agonism and antagonism identifies Orco as a promising target for the development of novel, broadly active insect repellants. Importantly, the ability of an Orco agonist to activate OSNs, as well as the ability of an antagonist to inhibit odorant-evoked OSN responses, have has been demonstrated *in vivo* using single sensillum recordings in *A. gambiae* [28,30]. Unfortunately, the currently identified Orco agonists and antagonists, such as OLC12 and OLC15 (this study), or VUAA1 [28] and VU0183254 [30], are unlikely to be useful as insect repellants due to low volatility. However, we have also shown that smaller structures, such as OLC20, are able to interact with Orco to inhibit activation of an OR complex by odorant. Thus, the pursuit of small, volatile agonists and antagonists of the Orco subunit may be a productive new phase of insect repellant development.

## Materials and Methods

### Materials

*Xenopus laevis* frogs were purchased from Nasco. The care and use of *X. laevis* frogs in this study were approved by the University of Miami Animal Research Committee and meet the guidelines of the National Institutes of Health. Odorants, Orco ligands and other chemicals were from Sigma-Aldrich, except for VUAA1, which was purchased from Vitas-M Laboratory, Ltd. (Apeldoorn, Netherlands) through eMolecules, Inc. (Solana Beach, CA). Dmel\Or35a and Dmel\Orco were generously provided by J. Carlson and L. Vosshall, respectively. Agam\Or65 and Agam\Orco, in pSP64T-Oligo and pT7TS, respectively [22], were generously provided by L. Zwiebel. Onub\Or1 and Onub\Orco were previously cloned [31], as were Cqui\Or10 and Cqui\Orco [25,26]. Dmel\Or35a, Dmel\Orco, Onub\Or1, Onub\Orco, Cqui\Or10 and Cqui\Orco were subcloned into pGEMHE [33].

### Expression of Insect ORs in *Xenopus* Oocytes

Oocytes were surgically removed from mature *Xenopus laevis* frogs. Follicle cells were removed by treatment with Collagenase B (Boehringer Mannhem) for 2 hours at room temperature. Capped cRNA encoding each DmOR subunit was generated using mMessage mMachine kits (Ambion). For heteromeric ORs, 25 ng of cRNA encoding each OR subunit was injected into Stage V-VI *Xenopus* oocytes. For expression of Orco alone, 50 ng of cRNA was injected. Oocytes were incubated at 18°C in Barth’s saline (in mM: 88 NaCl, 1 KCl, 2.4 NaHCO_3_, 0.3 CaNO_3_, 0.41 CaCl_2_, 0.82 MgSO_4_, 15 HEPES, pH 7.6, and 150 µg/ml ceftazidime) for 2–5 days prior to electrophysiological recording.

### Electrophysiology and Data Capture

Odorant and Orco ligand induced currents were recorded under two-electrode voltage clamp in *Xenopus laevis* oocytes expressing ORs, using an automated parallel electrophysiology system (OpusExpress 6000A; Molecular Devices). Oocytes were perfused with ND96 (in mM: 96 NaCl, 2 KCl, 1 CaCl_2_, 1 MgCl_2_, 5 HEPES, pH 7.5). Odorant stock solutions (0.5 or 1 M) and Orco ligand stock solutions (100 mM) were prepared in DMSO and diluted in ND96. Unless otherwise noted, applications were for 60 sec at a flow rate of 1.0 ml/min, with extensive washing in ND96 (9 or 20 min at 4.6 ml/min) between applications. Micropipettes were filled with 3 M KCl and had resistances of 0.2−2.0 MΩ. The holding potential was −70 mV. Current responses, filtered (4-pole, Bessel, low pass) at 20 Hz (−3 db) and sampled at 100 Hz, were captured and stored using OpusXpress 1.1 software (Molecular Devices).

### Experimental Protocols and Data Analysis

In inhibition assays, oocytes were exposed to 60 sec applications of OLC12 with 9 min washes between applications (Fig. 3 and 4) or 60 sec applications of odorant with 20 min washes between applications (Fig. 5). Oocytes were then exposed to a 90 sec application of antagonist candidate, immediately followed by a 60 sec co-application of antagonist candidate and OLC12 or odorant. Oocytes were then exposed to a final 60 sec application of OLC12 or odorant. Example traces illustrating this protocol are shown in Fig. 3A,B and 5A. The current response in the presence of antagonist candidate was compared to the preceding response(s) to OLC12 (or odorant) alone.

Initial analysis of electrophysiological data was done using Clampfit 9.1 software (Molecular Devices). Curve fitting and statistical analyses were done using Prism 5 (Graphpad). Concentration-response data were fit to the equation: I = I_max_/(1+(EC_50_/X)^n^) where I represents the current response at a given concentration of odorant, X; I_max_ is the maximal response; EC_50_ is the concentration of agonist yielding a half maximal response; n is the apparent Hill coefficient. Concentration-inhibition data were fit to the equation: I = I_max_/(1+(X/IC_50_)^n^) where I represents the current response at a given concentration of inhibitor, X; I_max_ is the maximal response in the absence of inhibitor; IC_50_ is the concentration of inhibitor present that still allows a half maximal response from odorant; n is the apparent Hill coefficient. Statistical significance was assessed using a two-tailed unpaired *t* test, an F test, or a one-way analysis of variance followed by the Bonferroni’s post-test, as appropriate.

## Supporting Information

Figure S1 **Sham (water injected) oocytes do not respond to Orco agonists and antagonists.** Compounds were applied for 60 seconds at a concentration of 100 µM, except for OLC15 which was applied at 50 µM. Each trace is representative of results from 4 oocytes.
(PDF)

Figure S2 **N-,2-substituted triazolothioacetamide compounds tested in this study.**
(PDF)

Figure S3 **Additional structures tested in this study.**
(PDF)

Figure S4 **Antagonism of Cqui\Orco.** Results of a screen of 8 compounds for Orco antagonism. Responses of Cqui\Orco + Cqui\Or10 to 3 µM OLC12 (∼EC25) (A) or of Cqui\Orco to 30 µM OLC12 (∼EC10) (B) in the presence of each candidate antagonist are presented as a percentage of the average of the two preceding responses to OLC12 alone (mean±SEM, n = 3−5).
(PDF)

Table S1 **Values from** **Figure 1C** **.** Response amplitudes to 100 µM of each compound are presented as a percentage of the response of the same oocyte to 100 µM OLC3 (mean±SEM, n = 3−8). nt, not tested.
(PDF)

## References

[1] Benton R, Vannice KS, Gomez-Diaz C, Vosshall LB (2009). Variant ionotropic glutamate receptors as chemosensory receptors in Drosophila.. Cell.

[2] Hallem EA, Dahanukar A, Carlson JR (2006). Insect odor and taste receptors.. Annu Rev Entomol.

[3] Vosshall LB, Stocker RF (2007). Molecular architecture of smell and taste in Drosophila.. Annu Rev Neurosci.

[4] Sato K, Pellegrino M, Nakagawa T, Vosshall LB, Touhara K (2008). Insect olfactory receptors are heteromeric ligand-gated ion channels.. Nature.

[5] Wicher D, Schafer R, Bauernfeind R, Stensmyr MC, Heller R (2008). Drosophila odorant receptors are both ligand-gated and cyclic-nucleotide-activated cation channels.. Nature.

[6] Vosshall LB, Hansson BS (2011). A unified nomenclature system for the insect olfactory coreceptor.. Chem Senses.

[7] Benton R, Sachse S, Michnick SW, Vosshall LB (2006). Atypical membrane topology and heteromeric function of Drosophila odorant receptors in vivo.. PLoS Biol.

[8] Jones WD, Nguyen TA, Kloss B, Lee KJ, Vosshall LB (2005). Functional conservation of an insect odorant receptor gene across 250 million years of evolution.. Curr Biol.

[9] Krieger J, Klink O, Mohl C, Raming K, Breer H (2003). A candidate olfactory receptor subtype highly conserved across different insect orders.. J Comp Physiol A Neuroethol Sens Neural Behav Physiol.

[10] Larsson MC, Domingos AI, Jones WD, Chiappe ME, Amrein H (2004). Or83b encodes a broadly expressed odorant receptor essential for Drosophila olfaction.. Neuron.

[11] Nakagawa T, Sakurai T, Nishioka T, Touhara K (2005). Insect sex-pheromone signals mediated by specific combinations of olfactory receptors.. Science.

[12] Neuhaus EM, Gisselmann G, Zhang W, Dooley R, Stortkuhl K (2005). Odorant receptor heterodimerization in the olfactory system of Drosophila melanogaster.. Nat Neurosci.

[13] Pitts RJ, Fox AN, Zwiebel LJ (2004). A highly conserved candidate chemoreceptor expressed in both olfactory and gustatory tissues in the malaria vector Anopheles gambiae.. Proc Natl Acad Sci U S A.

[14] Wetzel CH, Behrendt HJ, Gisselmann G, Stortkuhl KF, Hovemann B (2001). Functional expression and characterization of a Drosophila odorant receptor in a heterologous cell system.. Proc Natl Acad Sci U S A.

[15] Smart R, Kiely A, Beale M, Vargas E, Carraher C (2008). Drosophila odorant receptors are novel seven transmembrane domain proteins that can signal independently of heterotrimeric G proteins.. Insect Biochem Mol Biol.

[16] Nichols AS, Chen S, Luetje CW (2011). Subunit contributions to insect olfactory receptor function: channel block and odorant recognition.. Chem Senses.

[17] Pask GM, Jones PL, Rutzler M, Rinker DC, Zwiebel LJ (2011). Heteromeric anopheline odorant receptors exhibit distinct channel properties.. PLoS One.

[18] Nakagawa T, Pellegrino M, Sato K, Vosshall LB, Touhara K (2012). Amino Acid Residues Contributing to Function of the Heteromeric Insect Olfactory Receptor Complex.. PLoS ONE.

[19] Carey AF, Wang G, Su CY, Zwiebel LJ, Carlson JR (2010). Odorant reception in the malaria mosquito Anopheles gambiae.. Nature.

[20] Hallem EA, Carlson JR (2006). Coding of odors by a receptor repertoire.. Cell.

[21] Hallem EA, Ho MG, Carlson JR (2004). The molecular basis of odor coding in the Drosophila antenna.. Cell.

[22] Wang G, Carey AF, Carlson JR, Zwiebel LJ (2010). Molecular basis of odor coding in the malaria vector mosquito Anopheles gambiae.. Proc Natl Acad Sci U S A.

[23] Nichols AS, Luetje CW (2010). Transmembrane segment 3 of Drosophila melanogaster odorant receptor subunit 85b contributes to ligand-receptor interactions.. J Biol Chem.

[24] Pellegrino M, Steinbach N, Stensmyr MC, Hansson BS, Vosshall LB (2011). A natural polymorphism alters odour and DEET sensitivity in an insect odorant receptor.. Nature.

[25] Hughes DT, Pelletier J, Luetje CW, Leal WS (2010). Odorant receptor from the southern house mosquito narrowly tuned to the oviposition attractant skatole.. J Chem Ecol.

[26] Pelletier J, Hughes DT, Luetje CW, Leal WS (2010). An odorant receptor from the southern house mosquito Culex pipiens quinquefasciatus sensitive to oviposition attractants.. PLoS One.

[27] Xia Y, Wang G, Buscariollo D, Pitts RJ, Wenger H (2008). The molecular and cellular basis of olfactory-driven behavior in Anopheles gambiae larvae.. Proc Natl Acad Sci U S A.

[28] Jones PL, Pask GM, Rinker DC, Zwiebel LJ (2011). Functional agonism of insect odorant receptor ion channels.. Proc Natl Acad Sci U S A.

[29] Martin JP, Beyerlein A, Dacks AM, Reisenman CE, Riffell JA (2011). The neurobiology of insect olfaction: sensory processing in a comparative context.. Prog Neurobiol.

[30] Jones PL, Pask GM, Romaine IM, Taylor RW, Reid PR (2012). Allosteric antagonism of insect odorant receptor ion channels.. PLoS One.

[31] Wanner KW, Nichols AS, Allen JE, Bunger PL, Garczynski SF (2010). Sex pheromone receptor specificity in the European corn borer moth, Ostrinia nubilalis.. PLoS One.

[32] Bohbot JD, Dickens JC (2012). Odorant receptor modulation: Ternary paradigm for mode of action of insect repellents.. Neuropharm.

[33] Liman ER, Tytgat J, Hess P (1992). Subunit stoichiometry of a mammalian K+ channel determined by construction of multimeric cDNAs.. Neuron.

