# Supplementary material for: Identification of New Agonists and Antagonists of the Insect Odorant Receptor Co-Receptor Subunit
Source: PLoS One. 2012 May 8;7(5):e36784. doi: 10.1371/journal.pone.0036784 (PMC3348135; doi:10.1371/journal.pone.0036784)

Figure S1. Sham (water injected) oocytes do not respond to Orco agonists and antagonists. Compounds were applied for 60 seconds at a concentration of 100 μM, except for OLC15, which was applied at 50 μM. Each trace is representative of results from 4 oocytes.

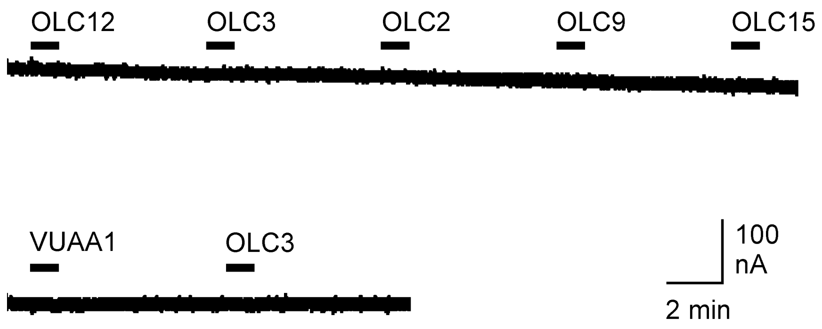

Supplement: Figure S1 — Sham (water injected) oocytes do not respond to Orco agonists and antagonists. Compounds were applied for 60 seconds at a concentration of 100 µM, except for OLC15 which was applied at 50 µM. Each trace is representative of results from 4 oocytes. (PDF) [file pone.0036784.s001.pdf]
