# Supplementary material for: Identification of New Agonists and Antagonists of the Insect Odorant Receptor Co-Receptor Subunit
Source: PLoS One. 2012 May 8;7(5):e36784. doi: 10.1371/journal.pone.0036784 (PMC3348135; doi:10.1371/journal.pone.0036784)
Supplement: Figure S2 — N-,2-substituted triazolothioacetamide compounds tested in this study. (PDF) [file pone.0036784.s002.pdf]

Figure S2.
N-,2-substituted triazolothioacetamide compounds tested in this study.

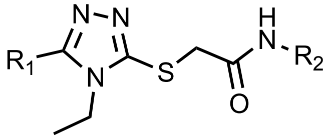

Compound Name	Screen Name	CAS #	R_1	R_2
N-(4-ethylphenyl)-2-((4-et-5-(3-pyridinyl)-4H-1,2,4-triazol-3-yl)thio)acetamide	VUAA1	525582-84-7	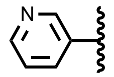	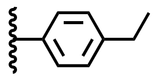
N-(4-ethylphenyl)-2-((4-et-5-(2-pyridinyl)-4H-1,2,4-triazol-3-yl)thio)acetamide	OLC2	577697-46-2	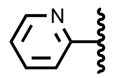	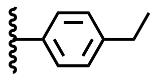
N-(4-ethylphenyl)-2-((4-et-5-(4-pyridinyl)-4H-1,2,4-triazol-3-yl)thio)acetamide	OLC3	618427-06-8	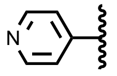	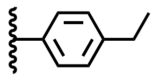
N-(4-ethylphenyl)-2-((4-et-5-(2-pyrazinyl)-4H-1,2,4-triazol-3-yl)thio)acetamide	OLC4	578723-85-0	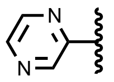	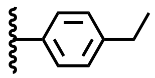
2-((4-Et-5-(3-pyridinyl)-4H-1,2,4-triazol-3-yl)thio)-N-(4-methylphenyl)acetamide	OLC5	333418-84-1	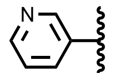	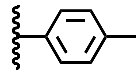
2-((4-Et-5-(3-pyridinyl)-4H-1,2,4-triazol-3-yl)thio)-N-(3-methylphenyl)acetamide	OLC6	333331-30-9	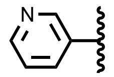	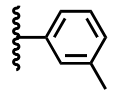
2-((4-Et-5-(3-pyridinyl)-4H-1,2,4-triazol-3-yl)thio)-N-(4-methoxy-ph)acetamide	OLC7	142529-68-8	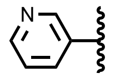	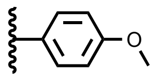
N-(2-ethylphenyl)-2-((4-et-5-(3-pyridinyl)-4H-1,2,4-triazol-3-yl)thio)acetamide	OLC8	142529-69-9	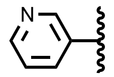	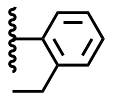
N-(4-butylphenyl)-2-((4-et-5-(3-pyridinyl)-4H-1,2,4-triazol-3-yl)thio)acetamide	OLC9	618426-72-5	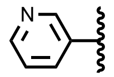	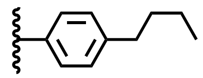
N-(4-br-ph)-2-((4-ethyl-5-(3-pyridinyl)-4H-1,2,4-triazol-3-yl)thio)acetamide	OLC10	142529-64-4	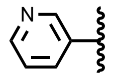	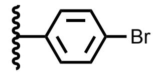
N-(4-cl-ph)-2-((4-ethyl-5-(3-pyridinyl)-4H-1,2,4-triazol-3-yl)thio)acetamide	OLC11	333331-28-5	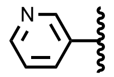	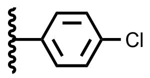
2-((4-Ethyl-5-(4-pyridinyl)-4H-1,2,4-triazol-3-yl)sulfanyl)-N-(4-isopropylphenyl)acetamide	OLC12	585550-72-7	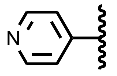	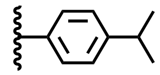
N-(3,4-dimethylphenyl)-2-((4-ethyl-5-(4-pyridinyl)-4H-1,2,4-triazol-3-yl)sulfanyl)acetamide	OLC13	482639-64-5	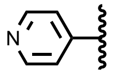	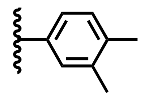
N-(4-(dimethylamino)phenyl)-2-((4-ethyl-5-(4-pyridinyl)-4H-1,2,4-triazol-3-yl)sulfanyl)acetamide	OLC14	618427-00-2	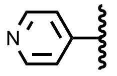	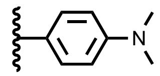
N-(4-butylphenyl)-2-((4-et-5-(2-pyridinyl)-4H-1,2,4-triazol-3-yl)thio)acetamide	OLC15	618416-06-1	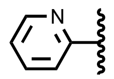	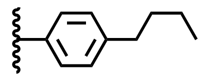
