# Supplementary material for: Identification of New Agonists and Antagonists of the Insect Odorant Receptor Co-Receptor Subunit
Source: PLoS One. 2012 May 8;7(5):e36784. doi: 10.1371/journal.pone.0036784 (PMC3348135; doi:10.1371/journal.pone.0036784)
Supplement: Figure S3 — Additional structures tested in this study. (PDF) [file pone.0036784.s003.pdf]

Figure S3. Additional structures tested in this study.

Compound Name	Screen Name	CAS #	Structure
5-(3-Pyridyl)-4H-1,2,4-triazole-3-thiol	OLC16	32362-88-2	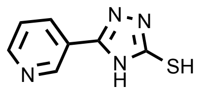
4-Allyl-5-pyridin-3-yl-4H-(1,2,4)triazole-3-thiol	OLC17		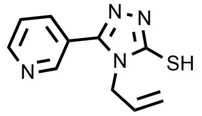
4-(4-Methyl-4H-1,2,4-triazol-3-yl)pyridine	OLC18		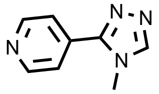
4'-Ethylacetanilide	OLC19	3663-34-1	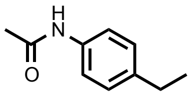
N-(4-ethylphenyl)-2-thiophenecarboxamide	OLC20		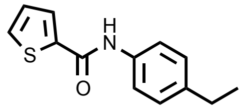
N-(4-isopropylphenyl)-2-[(1-methyl-1H-tetrazol-5-yl)sulfanyl]acetamide	OLC21		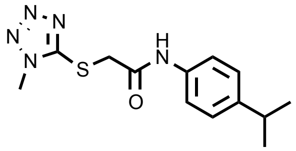
N-(4-butylphenyl)-2-thiophenecarboxamide	OLC22		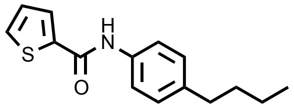
2-(5-Methyl-4H-1,2,4-triazol-3-yl)pyridine	OLC23		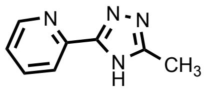
