# Supplementary material for: Identification of New Agonists and Antagonists of the Insect Odorant Receptor Co-Receptor Subunit
Source: PLoS One. 2012 May 8;7(5):e36784. doi: 10.1371/journal.pone.0036784 (PMC3348135; doi:10.1371/journal.pone.0036784)

Figure S4. Antagonism of Cqui\Orco. Results of a screen of 8 compounds for Orco antagonism. Responses of Cqui\Orco + Cqui\Or10 to 3 μM OLC12 (~EC_{25}) (**A**) or of Cqui\Orco to 30 μM OLC12 (~EC_{10}) (**B**) in the presence of each candidate antagonist are presented as a percentage of the average of the two preceding responses to OLC12 alone (mean ± SEM, n = 3-5).

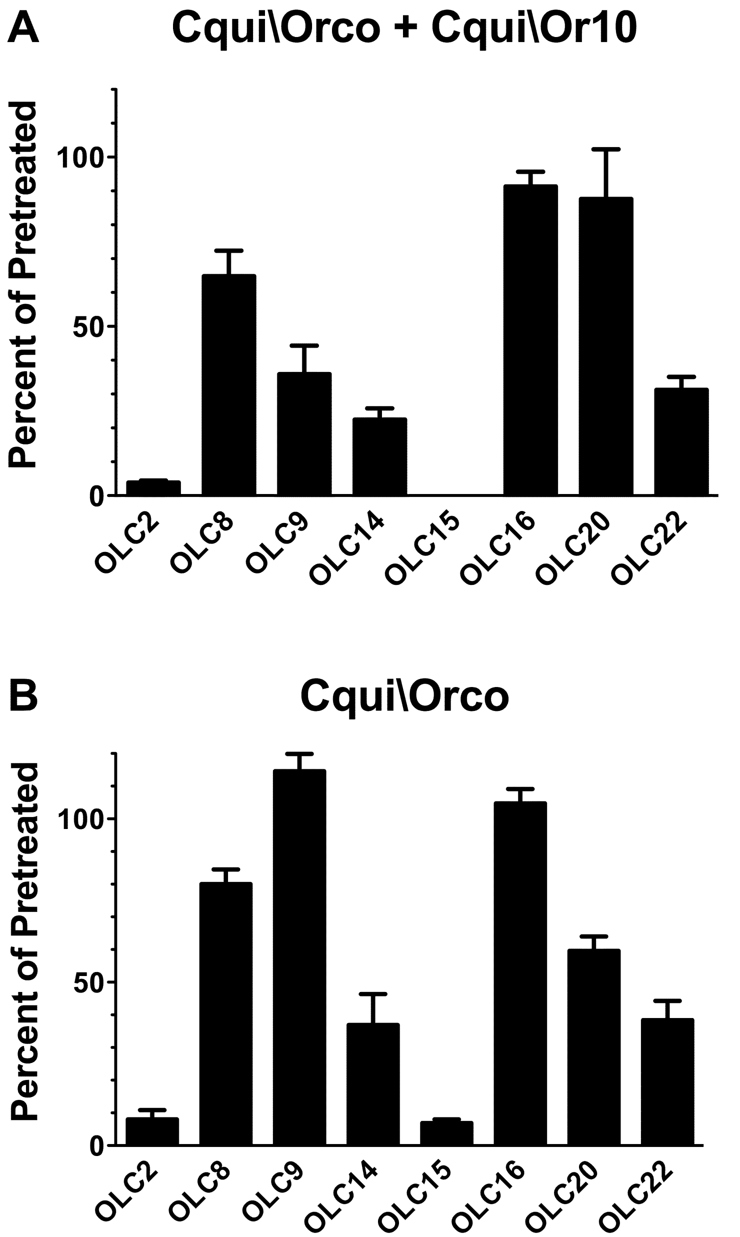

Supplement: Figure S4 — Antagonism of Cqui\Orco. Results of a screen of 8 compounds for Orco antagonism. Responses of Cqui\Orco + Cqui\Or10 to 3 µM OLC12 (∼EC25) (A) or of Cqui\Orco to 30 µM OLC12 (∼EC10) (B) in the presence of each candidate antagonist are presented as a percentage of the average of the two preceding responses to OLC12 alone (mean±SEM, n = 3−5). (PDF) [file pone.0036784.s004.pdf]
