# Supplementary material for: Identification of New Agonists and Antagonists of the Insect Odorant Receptor Co-Receptor Subunit
Source: PLoS One. 2012 May 8;7(5):e36784. doi: 10.1371/journal.pone.0036784 (PMC3348135; doi:10.1371/journal.pone.0036784)
Supplement: Table S1 — Values from Figure 1C . Response amplitudes to 100 µM of each compound are presented as a percentage of the response of the same oocyte to 100 µM OLC3 (mean±SEM, n = 3−8). nt, not tested. (PDF) [file pone.0036784.s005.pdf]

Table S1. Values from Figure 1C. Response amplitudes to 100 μM of each compound are presented as a percentage of the response of the same oocyte to 100 μM OLC3 (mean ± SEM, n=3-8). nt, not tested.

	Dmel\Orco + Dmel\Or35a	Dmel\Orco	Cqui\Orco + Cqui\Or10	Cqui\Orco	Onub\Orco + Onub\Or1
**VUAA1**	50 ± 9	24 ± 7	75 ± 3	18 ± 8	60 ± 6
**OLC2**	2 ± 1	0	0	0	9 ± 2
**OLC3**	100	100	100	100	100
**OLC4**	4 ± 1	0	0	0	4 ± 2
**OLC5**	4 ± 1	0	16 ± 2	0	4 ± 2
**OLC6**	0	0	0	4 ± 2	0
**OLC7**	4 ± 1	0	4 ± 1	4 ± 2	0
**OLC8**	0	0	0	5 ± 3	0
**OLC9**	5 ± 2	0	0	0	3 ± 1
**OLC10**	25 ± 7	0	27 ± 16	0	nt
**OLC11**	13 ± 2	0	13 ± 3	0	nt
**OLC12**	300 ± 41	318 ± 12	289 ± 41	636 ± 78	306 ± 33
**OLC13**	0	0	0	0	nt
**OLC14**	2 ± 1	0	12 ± 2	0	nt
**OLC15**	0	0	0	0	nt
**OLC16**	0	0	0	3 ± 2	nt
**OLC17**	0	0	0	3 ± 2	nt
**OLC18**	0	0	0	0	nt
**OLC19**	0	0	0	3 ± 2	nt
**OLC20**	0	0	11 ± 4	0	nt
**OLC21**	0	0	0	0	nt
**OLC22**	0	0	0	0	nt
**OLC23**	0	0	8 ± 3	0	nt
